# Zika Virus-Mediated Death of Hippocampal Neurons Is Independent From Maturation State

**DOI:** 10.3389/fncel.2019.00389

**Published:** 2019-08-27

**Authors:** Caroline Büttner, Maxi Heer, Jasmin Traichel, Martin Schwemmle, Bernd Heimrich

**Affiliations:** ^1^Department of Neuroanatomy, Institute of Anatomy and Cell Biology, Center for Basics in NeuroModulation, University of Freiburg, Freiburg, Germany; ^2^Faculty of Medicine, University of Freiburg, Freiburg, Germany; ^3^Institute of Virology, Medical Center University of Freiburg, Freiburg, Germany

**Keywords:** Zika virus, hippocampus, cell death, organotypic slice culture, electron microscopy

## Abstract

Zika virus (ZIKV) infection of pregnant women and diaplazental transmission to the fetus is linked to the congenital syndrome of microcephaly in newborns. This neuropathology is believed to result from significant death of neuronal progenitor cells (NPC). Here, we examined the fate of neurons in the developing hippocampus, a brain structure which houses neuronal populations of different maturation states. For this purpose, we infected hippocampal slice cultures from immunocompetent newborn mice with ZIKV and monitored changes in hippocampal architecture. In neurons of all hippocampal subfields ZIKV was detected by immunofluorescence labeling and electron microscopy. This includes pyramidal neurons that maturate during the embryonic phase. In the dentate gyrus, ZIKV could be found in the Cajal–Retzius (CR) cells which belong to the earliest born cortical neurons, but also in granule cells that are predominantly generated postnatally. Intriguingly, virus particles were also present in the correctly outgrowing mossy fiber axons of juvenile granule cells, suggesting that viral infection does not impair region- and layer-specific formation of this projection. ZIKV infection of hippocampal tissue was accompanied by both a profound astrocyte reaction indicating tissue injury and a microglia response suggesting phagocytotic activity. Furthermore, depending on the viral load and incubation time, we observed extensive overall neuronal loss in the cultured hippocampal slice cultures. Thus, we conclude ZIKV can replicate in various neuronal populations and trigger neuronal death independent of the maturation state of infected cells.

## Introduction

Zika virus (ZIKV), a member of the *Flavivirus* genus within the *Flaviviridae* family was identified almost 70 years ago in Africa, but severe diseases were not reported. The spread of ZIKV infections in 2015–2016 to South America, including Brazil, caused, however, a broad range of neurological symptoms in infected individuals and has brought this disease into world’s attention. Infection of pregnant women with ZIKV can lead to congenital transmission to the unborn child resulting in microcephaly (Kleber de Oliveira et al., 2016). More prevalent brain dysfunctions include abnormal limb postures and severe sensory defects. Furthermore, ZIKV infections are also associated with Guillain–Barré syndrome (Dos Santos et al., 2016).

Flaviviruses induce structural changes of the endoplasmic reticulum (ER) including convoluted membranes (CMs) and so-called vesicle packets (VPs), the presumed sites of viral RNA replication (Paul and Bartenschlager, 2013; Hamel et al., 2015). Using Huh7 human hepatic cells and human neuronal progenitor cells (NPC), a physiologically relevant target of ZIKV, Cortese et al. (2017) could show that ZIKV infection not only induces reorganization of the ER to form VPs and CMs but also affects intermediate filaments and microtubule network organelles to enable robust virus amplification. In return, preventing structural alterations by cytoskeleton stabilizing molecules suppresses ZIKV replication (Cortese et al., 2017).

Many studies focusing on ZIKV pathogenesis are performed using immunodeficient mouse lines (Lazear et al., 2016; Rossi et al., 2016), *in utero* infection of mice fetuses (Aliota et al., 2016; Miner et al., 2016; Yockey et al., 2016; Zhang et al., 2019) and *in vitro* approaches. In an organoid model of the developing brain it could be demonstrated that the Brazilian ZIKV strain H/PF/2013 (Asian lineage) causes apoptosis of NPC (Cugola et al., 2016; Dang et al., 2016). Similarly, Li C. et al. (2016) could show that infection with the African ZIKV strain MR766 is associated with neuron death, impaired cortical folding and expansion in cerebral organoids. A specific neural stem cell tropism for ZIKV was elucidated in embryonic cortical slice cultures (Brault et al., 2016). Neuronal death appears to be triggered by the release of tumor necrosis factor-α (TNF-α), interleukin-1β (IL-1β) and glutamate, which possess neurotoxic potential (Olmo et al., 2017). ZIKV infection not only occurs during early embryonic stages but also in later fetal/neonatal development stages of immunocompetent mice. After peripheral viral inoculation directly after birth, the virus enters the central nervous system (CNS) initially targeting astrocytes throughout the brain and subsequently neurons (van den Pol et al., 2017). Li H. et al. (2016) could demonstrate ZIKV induced neuronal death of stem cells in the neurogenic nische of hippocampus of adult mice. Moreover, unilateral microinjection of ZIKV into the brains of immunocompetent mice, resulted in infection of homotop contralateral cortical areas, indicating axonal transport of the virus to synaptically coupled brain regions. In our infection model, the hippocampus seems to be highly susceptible to ZIKV infection resulting in neuronal infection in the CA1 and CA3 regions already at 7 days post infection (p.i.), while infected neurons were prevalent in all examined brain areas at 14 days p.i. (Figure 3). At later stages of infection, beaded processes occur as signs of neuronal degeneration in some brain areas such as the hippocampus.

It remains unclear if neonate ZIKV infection perturbs normal development of the hippocampus, a brain structure involved in learning and memory. Laminar cytoarchitecture and connectivity patterns are characteristic features of the hippocampus and result from interplay of embryonic and postnatal neurogenesis, axon pathfinding and specific targeting. Organotypic hippocampal slice cultures from newborn immunocompetent mice differentiate morphological characteristics comparable to *in vivo* studies (Frotscher and Heimrich, 1993; Frotscher et al., 1995). Here, we provide evidence that neonate ZIKV infection does not perturb hippocampal development in mice but induces subsequent damage of neurons independent of their maturation state.

## Materials and Methods

### Preparation of Hippocampal Slice Cultures

Brains were removed from mice pups (P0–P2) and the hippocampi dissected under sterile conditions. Hippocampi were cut into 400 μm sections and transferred into petri dishes containing 4°C cold buffer solution consisting of minimal essential medium with a final concentration of 2 mM Glutamax at pH 7.3. Intact hippocampal slices were cultivated on porous Millipore membranes (Millicell Cell Culture inserts CM30org) in six well plates (4 cultures per well) filled with 1.2 ml nutrient medium (for details, see Mayer et al., 2005; Wu et al., 2013) for 7, 14, and 21 days. Medium was changed three times a week. Cultures were immediately infected after preparation with 1 μl of virus stock containing 10^5^ or 10^3^ PFU, respectively). For light and electron microscopic analysis *n* = 8 hippocampal cultures per experimental set were used.

### Immunofluorescence

Cultures selected for light microscopic analysis were fixed with 4% paraformaldehyde in 0.1 M phosphate buffer (PB), pH 7.3, for 2 h. After washing in PB cultures with the membrane underneath were cut off, mounted on a planar agar block, Vibratome resliced into 50 μm sections and collected in TBS. Prior immunolabeling sections were incubated in a blocking solution of 5% normal goat serum (NGS) and permeabilized in 0.3% Triton-X for 30 min. Primary antibodies (rabbit raised anti-calbindin (1: 5000, Swant); polyclonal anti-NeuN (1:1000, Abcam); rabbit-raised anti Iba1 (1:500, Wako)) for detecting microglial cells in PB containing 1% NGS + 0.1% Triton-X were applied and incubated at 4°C overnight. After several rinses in PB, sections were incubated with a secondary antibody (Cy3-conjugated goat anti-rabbit IgG, 1:1600; Alexa 488 conjugated goat anti rabbit IgG, 1:400, purchased from Dianova), respectively, for 3 h at room temperature in the dark. After rinsing in PB, sections were counterstained with DAPI (300 nM) for visualization of hippocampal cytoarchitecture. After several rinses in PB slices were mounted onto uncoated slides, embedded with Shandon Immu-mount (Thermo Fisher Scientific), coverslipped and digitally photographed with a Zeiss ApoTome 1. Cultures selected for light microscopic visualization of ZIKV were fixed and vibratome cut as described above. For permeabilization tissue was immersed in ice-cold MeOH for 20 min followed by incubation in TBS + 0.1% Triton-X for 30 min. Blocking solution was composed of 10% NGS and 1% Triton-X in TBS for 2.5 h. For double-immunofluorescence labeling a monoclonal mouse anti ZIKV antibody (Anti-Flavivirus group antigen [D1-4G2-4-15 (4G2)]; 1:300, Absolute Antibody) was used and combined with either calbindin or Iba1 staining (see above). As second layer for ZIKV immunofluorescence labeling Cy3-conjugated goat anti-mouse IgG (1:1600; Dianova) was applied.

### Electron Microscopy

Cultured hippocampal slices were immersed in a fixative solution (4% PFA, 0.05% glutaraldehyde) for 2 h post-fixed and washed in 50 mM TBS. Sections were osmicated, dehydrated, and flat-embedded in resin (Fluka Durcupan, EMS, Hatfield) on glass slides. Ultrathin sections (60 nm) were cut by an Ultracut (Leica EM UC7, Leica Biosystems) and collected on single-slot Formvar-coated nickel grids. Digital images were acquired using a transmission electron microscope (Leo 906 E, Carl Zeiss MicroImaging) equipped with a 2K sharp-eye CCD camera and processed with Image SysProg [Professional, version 1.2.5.118 (x64)] (Tröndle, Germany).

### Zika Virus Stocks

Virus stocks of the African Zika Virus strain (MR 766) were prepared in Vero cells and subsequently dialyzed in PBS for 12 h. Viral stock titers were determined by plaque forming units (PFU) in Vero cells as described (Nikolay et al., 2018).

### Western Blot Analysis

Membrane with cultures on top was washed with ice-cold PBS and the cultures were carefully removed. Four slice cultures were pooled, centrifuged (1,40,000 rpm) for 5 min at 4°C, resuspended in Ripa lysis buffer (Zurhove et al., 2008) and centrifuged again. Supernatant was transferred into non-reduced sample buffer (125 mM Tris–HCl, 20% glycerol, 4% SDS, 0.1 bromophenol blue) and heated up to 95°C for 5 min. Aliquots are stored at −80°C. For controls 15 μg/15 μl protein suspension were applied per line. Due to health safety reasons measurement of protein concentrations of ZIKV infected cultures was not performed. Protein extracts were size-fractionated by 15% SDS gel electrophoresis and blotted onto polyvinylidene difluoride (PVDF; 0.45 μm pore size, Millipore) for Western blot analysis. The membrane was blocked for 1 h in a solution consisting of 0.2% Tropix I-block plus 0.1% Tween-20 in TBS followed by incubation with primary antibodies against calbindin (1:1000, Swant), Iba1 (1:500 Wako), glial fibrillary acidic protein (GFAP; 1:1000, Cell Signaling), ZIKV (1:1000, Biozol) and a monoclonal antibody against β-actin (1:2000, Sigma) which serves as internal loading control of protein amount. After several rinses in TBS, the blot was incubated with alkaline phosphatase-conjugated secondary antibodies (1:10,000) for 45 min at RT. Proteins were visualized by chemiluminescence using CDP-star reagent kit according to the manufacturer’s instructions and Luminescent Image Analyzer Fuji Film IAS-3000. Densitometric analysis of images of western blots was carried out with the gel analysis plugin of Fiji software^1^.

## Results

**FIGURE 1:**
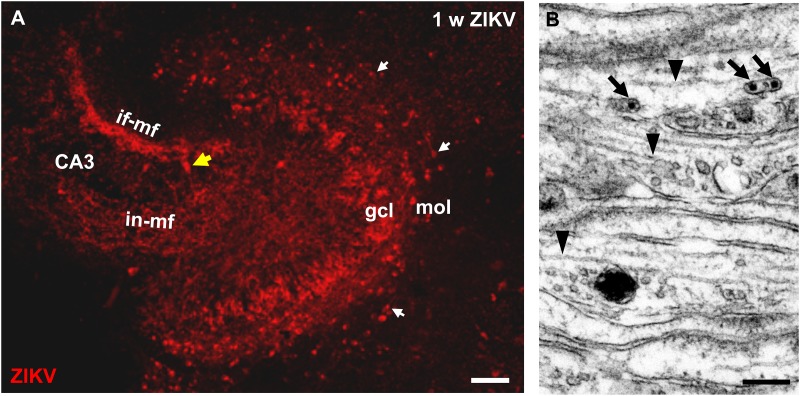
ZIKV is contained in mossy fiber axons of dentate granule cells. **(A)** Light microscopic image of a hippocampal culture immunostained with ZIKV-specific antibodies at 1 week p.i. ZIKV is detected in many somata of the granule cell layer (gcl). The granule cell axons give rise to a characteristic intra- and infrapyramidal mossy fiber projection toward CA3 region of the hippocampus. Horizontally oriented CR neurons (white arrows) in the molecular layer of the dentate gyrus also stain for ZIKV. Yellow arrow marks a ZIKV infected pyramidal neuron. **(B)** Electron micrograph of parallel running mossy fiber axons of a culture infected with Zika virus (10^5^ PFU) at 1 week p.i. Virions (arrows) are present inside an unmyelinated axon. Arrowheads mark intra-axonal microtubules. Scale bar: **(A)** 100 μm, **(B)** 200 nm.

To determine whether ZIKV infection affects normal development of the hippocampus, we infected organotypic hippocampal slice cultures from newborn immunocompetent mice. After infection of the cultures with 10^3^ PFU of ZIKV, virus-positive cells were detected by immunostaining in granule cells of the dentate gyrus 7 days p.i. (Figure 1A). Viral antigen was found in the typical c-shaped pattern of densely packed granule cell somata (Figure 1A). Of note, granule cell axons can be traced toward the CA3 region of the hippocampus by immunolabeling for ZIKV (Figure 1A). This axonal projection, the so-called mossy fiber tract which develops almost completely postnatal, displays the typical laminar appearance of an infra- and intrapyramidal portion both invading the CA3 hippocampal subfield (Figure 1A). This suggests that viral infection does not prohibit normal development of the mossy fiber pathway. Electron microscopy of the developing mossy fiber tract could confirm light microscopic indication of an intra-axonal localization of ZIKV. Small vesicular compartments containing viral particles could be found in parallel oriented and typically unmyelinated mossy fiber axons (Figure 1B). ZIKV infection is obviously not restricted to dentate granule cells of which the majority is born postnatally, since ZIKV-infected cells were observed in the hilus and CA3. Furthermore, in the molecular layer of the dentate gyrus i.e., above the ZIKV-positive granule cells, numerous horizontally oriented cells resembling the early generated Cajal–Retzius (CR) were found to be infected (Figure 1A).

**FIGURE 2:**
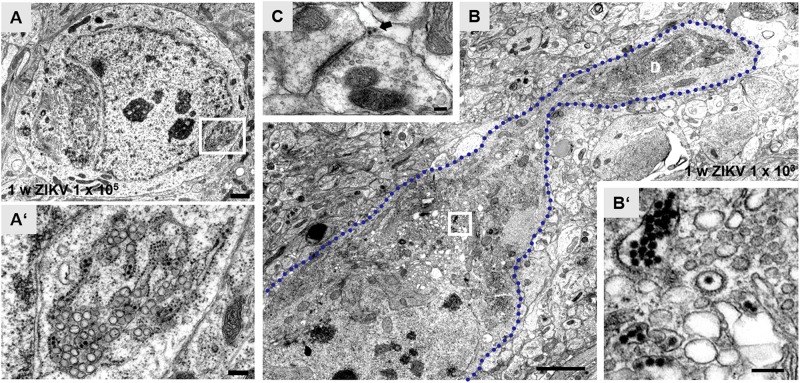
Zika virus-associated subcellular structural alterations of hippocampal principal neurons. **(A)** Electron micrograph of the soma of an infected dentate granule cell at 1 week p.i. with ZIKV (10^5^ PFU). The nucleus shows atypical invaginations and tubules of the endoplasmic reticulum (ER). **(A’)** High magnification of boxed area in panel **(A)**. Dilate rER cisternae contain virions (black arrows) and ZIKV-induced vesicles. Vacuoles (white arrow) are juxtaposed to ER. The bi-layered nuclear membrane (arrowheads) is obviously intact. **(B)** Example of an infected CA1 pyramidal neuron with a proximal dendritic segment. The blue dotted line outlines the cellular membrane of this CA1 neuron at 1 week p.i. with ZIKV (10^3^ PFU). Large empty vacuoles are easily visible in this overview image. **(B’)** High power magnification of the white box in panel **(B)** reveals clusters of ZIKV inside the rER and inside so-called vesicle packets. **(C)** Example of a presynaptic terminal contacting a postsynaptic spine at 1 week p.i. with ZIKV (10^3^ PFU). The arrow marks virus particles at the synaptic cleft. Scale bar: **(A)** 1 μm, **(A’)** 200 nm, **(B)** 2 μm, **(B’)** 100 nm, **(C)** 150 nm.

Using an infection dose of 10^5^ PFU of ZIKV, ultrastructural changes of hippocampal neurons can be readily observed 7 days p.i., including granule cells which developed atypical impressions of the nucleus (Figures 2A,A’). Opposite to these impressions, aggregates of ER are found. The cisternae often dilate, containing viral particles and are adjoined clusters of vacuoles (Figures 2A,A’). Similar morphological alterations of these cellular organelles even occur in infected pyramidal neurons that are reliably recognizable due to their localization and orientation inside the principal cell layer of the hippocampus with an apical stem dendrite extending into the stratum radiatum. Figure 2B shows the soma and a proximal dendrite of a CA1 pyramid that had been infected with 10^3^ PFU/ml. Again, vacuoles of different sizes are localized adjacent to ER cisternae. Viral particles are contained both inside the ER and vacuoles (Figures 2B,B’). Virus is found also in the vicinity of synaptic structures (Figure 2C).

**FIGURE 3:**
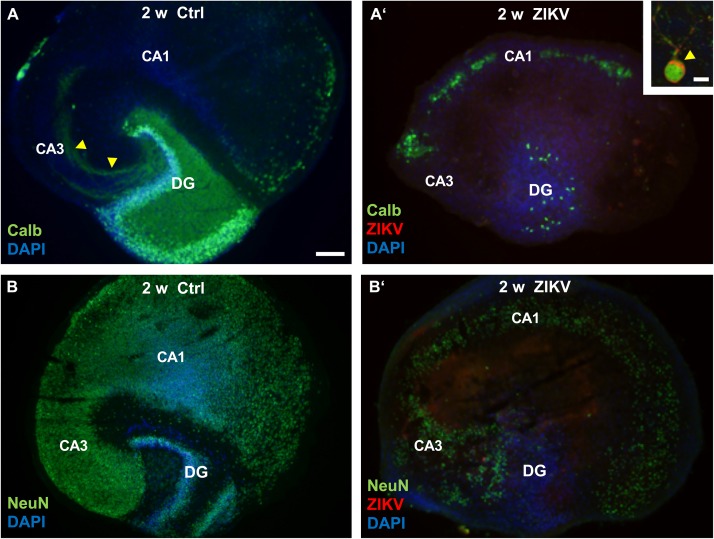
Neuronal loss in ZIKV-infected hippocampal cultures. **(A)** At DIV 14 a control culture displays normal hippocampal cytoarchitecture. Calbindin-immunofluorescence (green) labeling shows an intense labeling of granule cells expanding their dendrites into the molecular layer of the dentate gyrus as well as a typical mossy fiber projection (yellow arrowheads). **(A’)** Survey of a ZIKV-infected slice culture 14 days p.i. Note the dramatic decrease of calbindin-immunoreactivity and a dentate gyrus lacking typical laminar organization as indicated by DAPI nuclear stain (blue). Inset: Example of a calbindin-immunoreactive granule cell that is obviously infected by ZIKV (red). The arrowhead points to the strong immune-signal at the apical pole of this neuron. Green fluorescence at the rim of the cultures results from unspecific labeling. **(B)** Example of a non-infected hippocampal culture immunostained with an antibody against NeuN (green), a nuclear marker for mature neurons. C-shaped granule cell layer (DG) and the cell layers of the hippocampal subfields CA3 and CA1 are clearly visible. **(B’)** NeuN immunofluorescence is remarkably reduced in all hippocampal areas of an infected culture at same incubation time. DAPI counterstaining shows scattered cellular pattern of the dentate gyrus. CA3: regio inferior, CA1: regio superior, DG: dentate gyrus. Scale bars: **(A,A’,B,B’)** 150 μm, inset: 10 μm.

After 2 weeks the non-infected hippocampal cultures display a normal cytoarchitecture (Figure 3A). The mature granule cells detected by calbindin staining constitute both the granule cell layer and the molecular layer, the latter mainly composed by granule cell dendrites. Furthermore, the mossy fiber projection terminates specifically in the CA3 region. Similar to *in vivo*, some neurons of CA1 and subiculum also express the calcium binding protein calbindin. In contrast, cultures infected with 10^3^ PFU/ml of ZIKV lack an intact granule cell layer and DAPI nuclear stain reveals a lack of laminar cellular organization of the dentate gyrus with only a few calbindin immuno-positive granule cells 2 weeks p.i. In addition, the intrinsic hippocampal mossy fiber projection has disappeared, suggesting massive granule cell death. Double labeling for calbindin and ZIKV enabled us to phenotypically identify remaining cells in the dentate gyrus as infected granule cells (Figure 3A’, inset). Next, we were asking if pyramidal neurons that are born at early embryonic stages and has differentiated prior to ZIKV infection, undergo apoptosis/cell death. By the use of immunofluorescence staining against NeuN a nuclear protein of mature neurons, uninfected hippocampal slice cultures show a strong immunoreactivity of a c-shaped granule cell layer and the cell layers of the CA3 and CA1 regions (Figure 3B). The broadening of cell layers *in vitro* is often observed in slice cultures due to flattening of the tissue during cultivation. Similar to the decrease in calbindin immunofluorescence in the dentate gyrus, infection with ZIKV results in a pronounced reduction in NeuN immunoreactivity in all hippocampal subfields indicating an overall demise/damage of different neuronal populations (Figure 3B’). As expected, infection with 10^5^ PFU of ZIKV caused a total disintegration of tissue in almost all cultured hippocampi (*n* = 8 per group) in less than 2 weeks *in vitro* preventing further histological analysis (data not shown).

**FIGURE 4:**
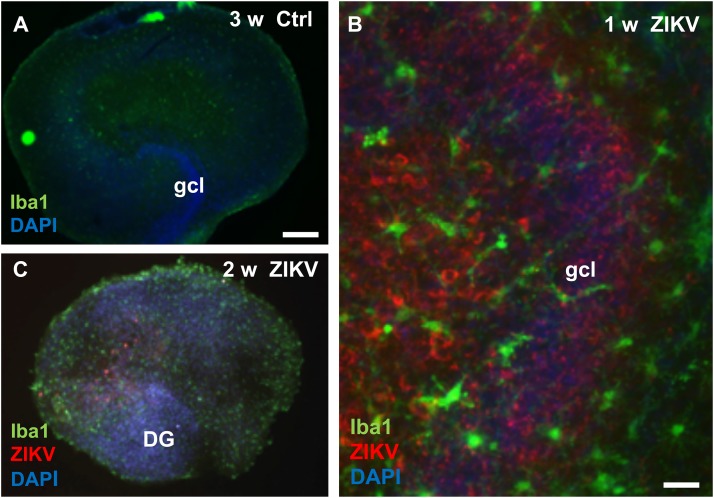
Microglia reaction in Zika virus infected hippocampal slice cultures. **(A)** At 3 weeks *in vitro* a non-infected hippocampal section shows only faint immunoreactivity for the microglia marker protein Iba1. **(B)** High magnification of an infected culture (10^3^ PFU) 7 days p.i. displays many ZIKV–immunopositive (red) cells, both in the granule cell layer and in the hilus. Of note, the granule cells are densely packed and form a normal granule cell layer (gcl). Iba1 immuno-stained microglia cells with branched processes (green) are often located in vicinity of ZIKV infected neuronal elements. **(C)** Micrograph of a hippocampal culture infected with 10^3^ PFU of ZIKV shows 14 days p.i. a pathological disorganization of the dentate gyrus and an increased Iba1 immunofluorescence already. Scale bar: **(A,B)** 300 μm, **(C)** 50 μm.

To investigate viral infection and a possible microglia response we compared Iba1, a marker for microglia cells, immunofluorescence pattern and intensity between uninfected controls and hippocampal slices infected with ZIKV at various cultivation periods. As shown exemplarily for a 21 day old culture, non-infected hippocampal slices display their typical cellular organization and a faint Iba1 signal (Figure 4A). In contrast, hippocampal cultures infected with ZIKV (10^3^ PFU) revealed intense Iba1 immunofluorescence labeling and numerous ZIKV-infected cells of the dentate gyrus and the hilar region were detected (Figure 4B). Moreover, the Iba1 immunofluorescence microglia processes are often closely positioned to ZIKV-infected cells (Figure 4B). Cultures infected for 2 weeks display an obvious loss of granule cell layer organization but intense Iba1 signals (Figure 4C).

**FIGURE 5:**
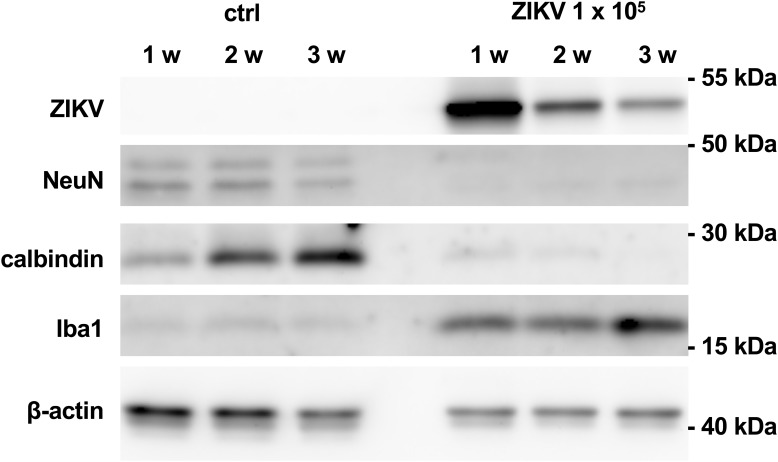
Viral and cellular protein levels in non-infected and ZIKV-infected hippocampal cultures. Hippocampal slice cultures (*n* = 4) were mock infected or infected with ZIKV (10^5^ PFU). After the indicated time points p.i., viral (E protein) and cellular protein levels (NeuN, calbindin, Iba1, and ß-actin) were determined by Western blot analysis.

To monitor loss of hippocampal cytoarchitecture and time-dependent damage of neurons, we performed a time course analysis of protein levels of non-infected cultures and cultures infected with 10^3^ PFU of ZIKV. Using this viral concentration, cultured hippocampi could be processed for western blot analysis even at an extended incubation period of 21 days p.i. (Figure 5), but not for further histological examination due to the extensive damage of the tissue. Infected hippocampi express viral proteins at all examined time points. However, the strongest signal was observed 7 days p.i. and declined at later stages. This reduction might reflect neuronal death of the ZIKV-infected cells. Protein levels for Iba1 are low in control cultures but high in infected cultures and increasing over incubation time. As expected, protein levels for calbindin remarkably increases in the control hippocampi during incubation period indicating maturation of dentate granule cells. This is in clear contrast to infected cultures. At 7 and 12 days p.i. the calbindin levels are low and completely vanish 21 days p.i. This suggests that ZIKV infection exerts deleterious effects on granule cells already at short infection periods. Likewise, NeuN protein levels are similar in control cultures with a slight decrease in cultures kept for 21 days, while NeuN protein amounts of infected slices are already low at 1 week p.i. Thus, a general death of both granule cells and neurons occurs in infected slice cultures. Using an identical experimental setting we compared activation of astrocytes by measuring GFAP levels as an additional parameter of brain tissue injury. Control cultures display a GFAP signal known as common feature due to the mechanical dissection of the hippocampi (del Rio et al., 1991). The signal intensity of GFAP stays similar at all examination time points (Supplementary Figure S1). In contrast ZIKV-infected cultures show enhanced GFAP levels already at 1 week p.i. with increasing protein content at prolonged cultivation of hippocampi. Comparing of GFAP signal intensity that had been corrected for internal β-actin load indicates to an about fourfold increase of GFAP level at 2 weeks p.i. (Supplementary Figure S1). This observation clearly points to enhanced activation of astrocytes in relation to progredient injury of ZIKV-infected hippocampal tissue.

## Discussion

The hippocampus is a highly organized cortical structure and neurons can be easily identified based upon characteristic morphology, protein expression patterns and their topography in the various hippocampal subfields. Due to the fact, that these features are maintained in long-term hippocampal slice cultures (Gähwiler, 1984; Frotscher et al., 1995), this *ex vivo* approach has been successfully used for investigating virus mediated neuronal pathologies (Mayer et al., 2005; Wu et al., 2013). Here, we can show that neurons of hippocampal slice cultures are highly susceptible for ZIKV infection, express morphological signs of viral replication and undergo neuronal death in a time dependent manner. At 7 days p.i. only very few pyramidal cells are ZIKV-infected in contrast to cells of the dentate gyrus. Especially granule cells and supragranular located CR cells of the marginal zone of the dentate gyrus seem to be highly susceptible to ZIKV infection. This suggests that neurons are the initial targets of ZIKV, which is in contrast to an initial strong infection of astrocytes followed by a transmission to neurons in the CNS of newborn mice (van den Pol et al., 2017).

Neuronal cell types of the hippocampus differ with respect to the date of birth and maturation at onset of ZIKV infection. CR neurons are among the first-born neurons during cortical development and contribute to cortical layering while the majority of granule cells are born postnatally (Bayer and Altman, 1990). In addition, a poor infection rate of pyramidal neurons which are generated prenatally, suggests cell type specific differences for ZIKV infection. In our study, we could trace a ZIKV immunoreactive mossy fiber projection emerging from dentate granule cells and terminating in the CA3 region of the hippocampus. This projection develops during the first postnatal weeks, both, *in vivo* and in hippocampal slice cultures and is characterized by its laminar trajectory and region-specific termination (Heimrich and Frotscher, 1991). Moreover, ZIKV-immunoreactive mossy fiber projections can be typically subdivided into an intra- and infrapyramidal portion with extension of the infrapyramidal projection to the CA3/CA1 border. This indicates that ZIKV does not impair outgrowth and target recognition of mossy fiber axons. With high resolution electron microscopy, we could also prove the presence of virions inside unmyelinated mossy axon bundles displaying both normal microtubular elements and organization, although changes in conformation of intermediate filaments and microtubules induced by neighboring viral particles have been reported in other cell systems (Cortese et al., 2017).

Both in granule cells and in pyramidal neurons, remodeling of ER occurs and cisternae often dilate, containing membranous invaginations, some of them filled with clusters of virus. These ZIKV induced so-called VPs are regarded as presumptive sites of viral RNA replication (Hamel et al., 2015; Cortese et al., 2017). Virions juxtaposed to the synaptic cleft of an excitatory terminal (Figure 2) point to viral release from infected hippocampal neurons.

With ongoing incubation periods, structural changes become apparent in ZIKV infected hippocampal cultures. Almost all hippocampi that had been infected with 10^5^ PFU of ZIKV displayed a complete loss of cytoarchitecture and could not be kept *in vitro* for more than 10 days impeding further histological examination. However, hippocampi infected with 10^3^ PFU allowed further analyses and revealed that NeuN and calbindin immunoreactivity was massively reduced and showed a disorganization of the dentate gyrus. This indicates neuronal damage in contrast to the normal cytoarchitecture and typical dentate-hippocampal interconnectivity in non-infected hippocampi similar to previous observations (Heimrich and Frotscher, 1991). Thus, we can demonstrate a time- and dose-dependent virus mediated damage of identified neuronal cell types in an organotypic brain culture. As expected, these observations of a progressive brain tissue injury after ZIKV infection are substantiated by a pronounced astrocyte activation.

In line, infection of hippocampal slices with ZIKV induces an activation of microglia. Processes of microglia cells touching ZIKV-immunoreactive structures suggest that microglia are employed in eliminating dead or infected cells. This may explain a progressive decline in protein levels for the neuronal markers calbindin and NeuN as well as for ZIKV over time.

In summary, our data show that ZIKV infection can occur in various neuronal cell types of the hippocampus at postnatal stage. This is in line with observations by Bell et al. (1971) after intracerebral ZIKV infection and strengthens the validity of the slice culture model. Efficient infection of neurons identifies this cell population as initial targets of ZIKV in the hippocampus. The simultaneous infection of neurons that are born prenatally and of immature neurons that are born postnatally indicate that susceptibility is independent of differentiation state. Thus, the observed delayed infection of already mature pyramidal neurons led to the assumption that other factors than differentiation state determine susceptibility/vulnerability.

## Ethics Statement

This study was carried out in accordance with the guidelines of the local authorities (RP Freiburg).

## Author Contributions

BH and MS designed the experiments and wrote the manuscript. CB, MH, JT, and BH performed the experiments.

## Conflict of Interest Statement

The authors declare that the research was conducted in the absence of any commercial or financial relationships that could be construed as a potential conflict of interest.

## Footnotes

1 https://fiji.sc/

## Supplementary Material

FIGURE S1 Comparison of GFAP protein values of one single experiment in non-infected and ZIKV-infected hippocampal cultures at different incubation time points. **(A)** control cultures (*n* = 4, each group) exhibit a similar GFAP expression at all time points. Note the increase of GFAP signals in infected cultures (*n* = 4, each group) already at 1 week of ZIKV infection. β-actin which serves as internal loading control is similarly low in infected cultures. **(B)** quantification of increasing GFAP levels in ZIKV-infected cultures by estimation GFAP/β-actin ratio.
