# Supplementary figures and images for: Zika Virus-Mediated Death of Hippocampal Neurons Is Independent From Maturation State
Source: Front Cell Neurosci. 2019 Aug 27;13:389. doi: 10.3389/fncel.2019.00389 (PMC6736629; doi:10.3389/fncel.2019.00389)

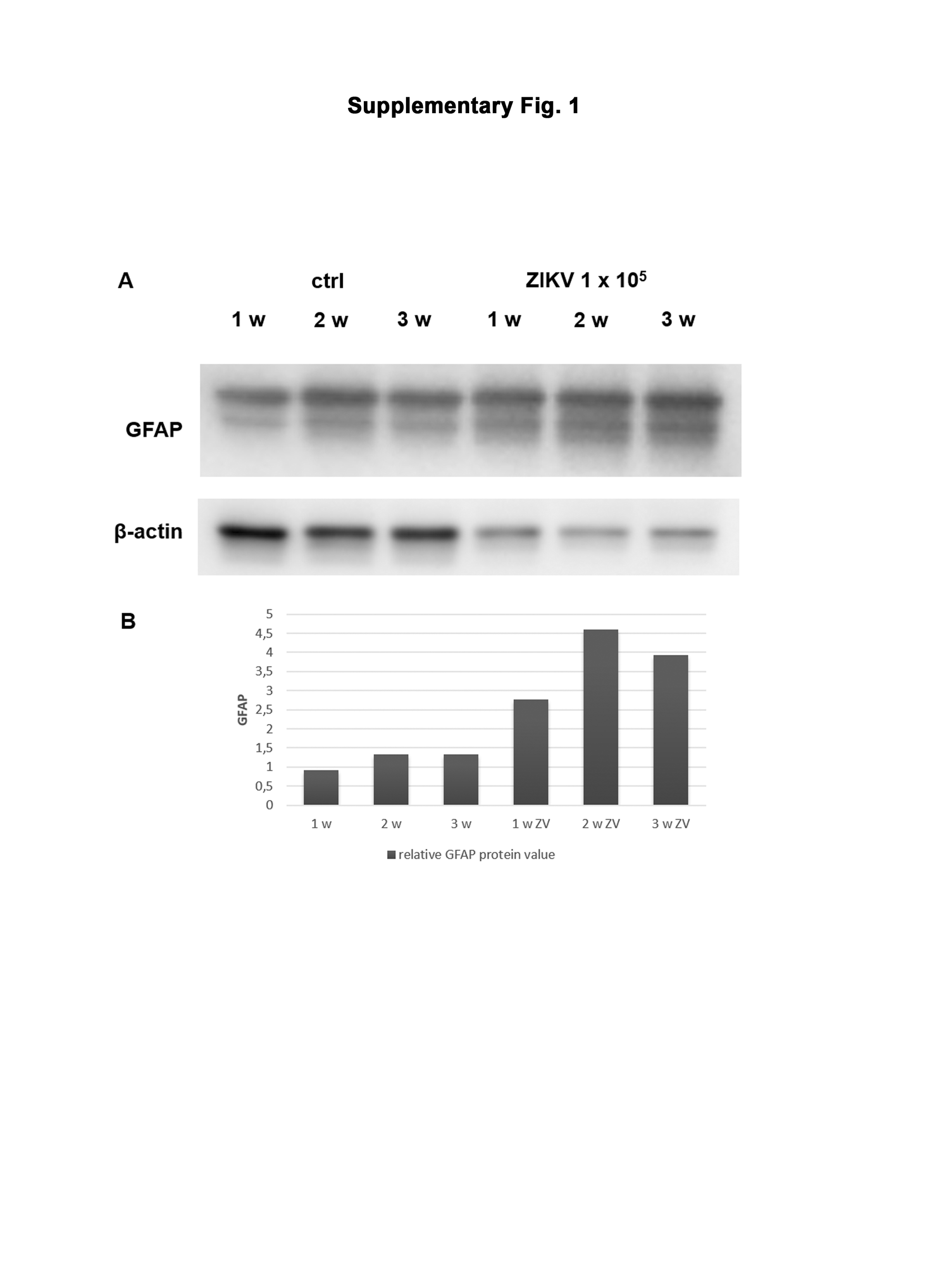

Supplement: FIGURE S1 — Comparison of GFAP protein values of one single experiment in non-infected and ZIKV-infected hippocampal cultures at different incubation time points. (A) control cultures (n = 4, each group) exhibit a similar GFAP expression at all time points. Note the increase of GFAP signals in infected cultures (n = 4, each group) already at 1 week of ZIKV infection. β-actin which serves as internal loading control is similarly low in infected cultures. (B) quantification of increasing GFAP levels in ZIKV-infected cultures by estimation GFAP/β-actin ratio. [file Image_1.tif]
